# Supplementary material for: Application of Metagenomic Next-Generation Sequencing in the Diagnosis of Pneumonia Caused by Chlamydia psittaci
Source: Microbiol Spectr. 2022 Aug 8;10(4):e02384-21. doi: 10.1128/spectrum.02384-21 (PMC9431268; doi:10.1128/spectrum.02384-21)
Supplement: Supplemental file 1 — Supplemental material. Download spectrum.02384-21-s0001.pdf, PDF file, 0.7 MB [file spectrum.02384-21-s0001.pdf]

BLAST Results

Questions/comments

Job title: Nucleotide Sequence

**RID**	AF0RH400013 (Expires on 05-22 17:52 pm)		
**Query ID**	lcl|Query_46425	**Database Name**	refseq_genomes (2 databases)
**Description**	None	**Description**	**Program** BLASTN 2.11.0+
**Molecule type**	dna		
**Query Length**	364		

Graphic Summary

Distribution of the top 54 Blast Hits on 54 subject sequences

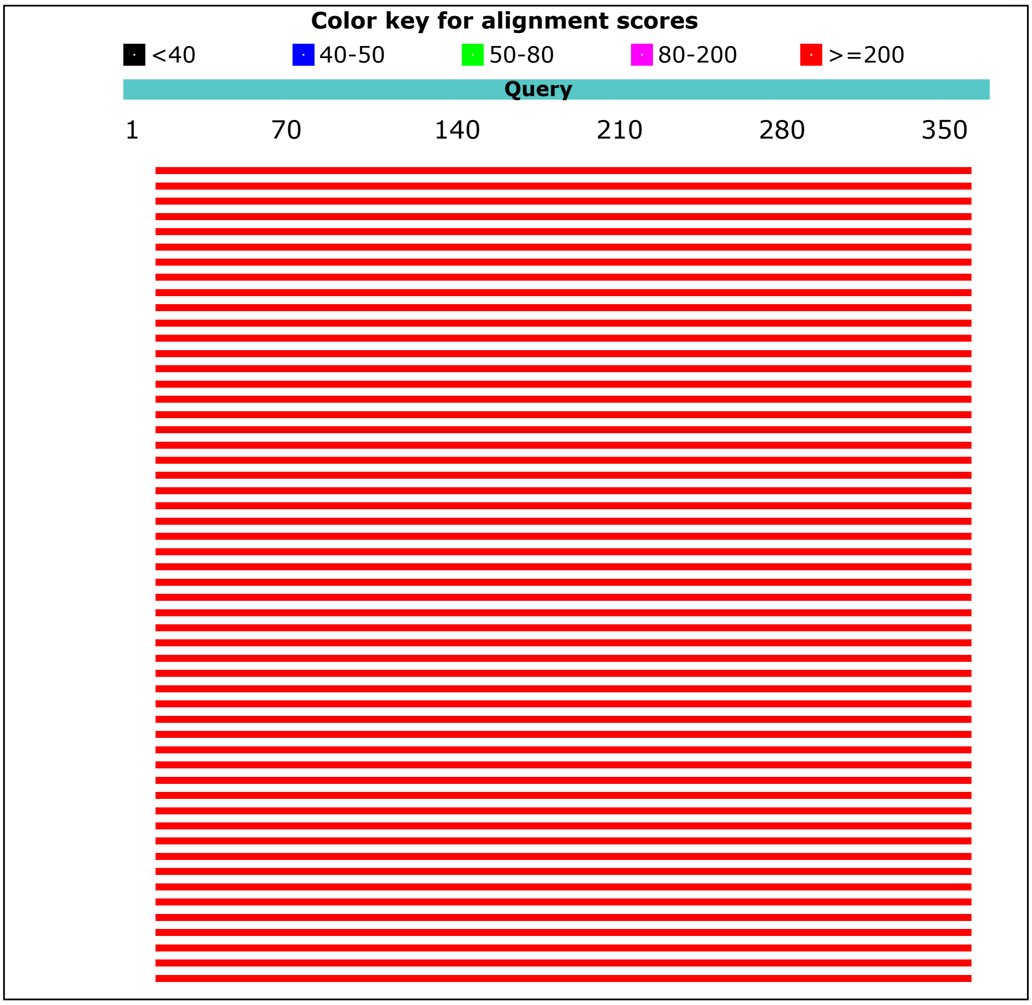

Descriptions

Sequences producing significant alignments:

Description	Max Score	Total Score	Query Cover	E value	Per. Ident	Accession
Chlamydia psittaci 6BC, complete sequence	645	645	95%	0.0	100.00%	NC_017287.1
Chlamydia psittaci 6BC, complete sequence	645	645	95%	0.0	100.00%	NC_015470.1
Chlamydia psittaci 08DC60 CP_08DC60.contig.15, whole genome shotgun sequence	645	645	95%	0.0	100.00%	NZ_KE355942.1
Chlamydia psittaci strain Fa An contig0013, whole genome shotgun sequence	645	645	95%	0.0	100.00%	NZ_LZRZ01000013.1
Chlamydia psittaci strain Zo Pa contig0003, whole genome shotgun sequence	645	645	95%	0.0	100.00%	NZ_LZRY01000003.1
Chlamydia psittaci strain CR009 contig0004, whole genome shotgun sequence	645	645	95%	0.0	100.00%	NZ_LZRX01000004.1
Chlamydia psittaci strain Fr Da contig0005, whole genome shotgun sequence	645	645	95%	0.0	100.00%	NZ_LZSA01000005.1
Chlamydia psittaci C6/98 CP_C6_98.contig.1, whole genome shotgun sequence	645	645	95%	0.0	100.00%	NZ_KE359922.1
Chlamydia psittaci 02DC24 CP_02DC24.contig.72, whole genome shotgun sequence	645	645	95%	0.0	100.00%	NZ_KE359643.1
Chlamydia psittaci strain 10652_placenta H5__contig_12, whole genome shotgun sequence	645	645	95%	0.0	100.00%	NZ_PJPX01000001.1
Chlamydia psittaci strain 8882_foetus NODE_7, whole genome shotgun sequence	645	645	95%	0.0	100.00%	NZ_PJPZ01000009.1
Chlamydia psittaci strain 8882_placenta H2_13, whole genome shotgun sequence	645	645	95%	0.0	100.00%	NZ_PJQA01000012.1
Chlamydia psittaci strain 9945_foetus H3_12, whole genome shotgun sequence	645	645	95%	0.0	100.00%	NZ_PJPY01000006.1
Chlamydia psittaci strain Ho Re lower contig0005, whole genome shotgun sequence	645	645	95%	0.0	100.00%	NZ_LZRF01000005.1
Chlamydia psittaci strain Ho Re upper contig0003, whole genome shotgun sequence	645	645	95%	0.0	100.00%	NZ_LZRE01000003.1
Chlamydia psittaci strain Horse_pl chromosome	645	645	95%	0.0	100.00%	NZ_CP025423.1
Chlamydia psittaci 02DC15, complete sequence	645	645	95%	0.0	100.00%	NC_017292.1

Description	Max Score	Total Score	Query Cover	E value	Per. Ident	Accession
Chlamydia psittaci C19/98, complete sequence	645	645	95%	0.0	100.00%	NC_017291.1
Chlamydia psittaci 08DC60, complete sequence	645	645	95%	0.0	100.00%	NC_017290.1
Chlamydia psittaci 01DC11, complete sequence	645	645	95%	0.0	100.00%	NC_017289.1
Chlamydia psittaci strain CB3 contig4, whole genome shotgun sequence	645	645	95%	0.0	100.00%	NZ_JMEI01000004.1
Chlamydia psittaci strain CB7 contig34, whole genome shotgun sequence	645	645	95%	0.0	100.00%	NZ_JMBZ01000034.1
Chlamydia psittaci UGA Contig04, whole genome shotgun sequence	645	645	95%	0.0	100.00%	NZ_AWXQ01000004.1
Chlamydia psittaci DD34 Contig01, whole genome shotgun sequence	645	645	95%	0.0	100.00%	NZ_AFVL01000001.1
Chlamydia psittaci Cal10 cal10.assembly.2, whole genome shotgun sequence	645	645	95%	0.0	100.00%	NZ_AEZD01000002.1
Chlamydia psittaci 02DC16 CP_02DC16.contig.1, whole genome shotgun sequence	645	645	95%	0.0	100.00%	NZ_KE353614.1
Chlamydia psittaci 02DC15 CP_02DC15.contig.1, whole genome shotgun sequence	645	645	95%	0.0	100.00%	NZ_KE353591.1
Chlamydia psittaci 01DC11 CP_01DC11.contig.0, whole genome shotgun sequence	645	645	95%	0.0	100.00%	NZ_KE353283.1
Chlamydia psittaci 03DC35 CP_03DC35.contig.40, whole genome shotgun sequence	645	645	95%	0.0	100.00%	NZ_KE356211.1
Chlamydia psittaci C19/98 CP_C19_98.contig.1, whole genome shotgun sequence	645	645	95%	0.0	100.00%	NZ_KE356193.1
Chlamydia psittaci 04DC42 CP_04DC42.contig.58, whole genome shotgun sequence	645	645	95%	0.0	100.00%	NZ_KE355923.1
Chlamydia psittaci 02DC23 CP_02DC23.contig.0, whole genome shotgun sequence	645	645	95%	0.0	100.00%	NZ_KE355767.1
Chlamydia psittaci 02DC22 CP_02DC22.contig.178, whole genome shotgun sequence	645	645	95%	0.0	100.00%	NZ_KE355746.1
Chlamydia psittaci 02DC21 CP_02DC21.contig.0, whole genome shotgun sequence	645	645	95%	0.0	100.00%	NZ_KE355432.1
Chlamydia psittaci 02DC18 CP_02DC18.contig.0, whole genome shotgun sequence	645	645	95%	0.0	100.00%	NZ_KE353617.1
Chlamydia psittaci RD1, complete sequence	645	645	95%	0.0	100.00%	NC_014796.1

Description	Max Score	Total Score	Query Cover	E value	Per. Ident	Accession
Chlamydia psittaci strain Po An contig0004, whole genome shotgun sequence	645	645	95%	0.0	100.00%	NZ_LZRG01000004.1
Chlamydia psittaci strain GIMC 2003:Cps25SM chromosome, complete genome	645	645	95%	0.0	100.00%	NZ_CP024453.1
Chlamydia psittaci strain GIMC 2005:CpsCP1 chromosome, complete genome	645	645	95%	0.0	100.00%	NZ_CP024451.1
Chlamydia psittaci strain GIMC 2004:CpsAP23 chromosome, complete genome	645	645	95%	0.0	100.00%	NZ_CP024455.1
Chlamydia psittaci 99DC5 CP_99DC5.contig.63, whole genome shotgun sequence	628	628	95%	1e-179	99.14%	NZ_KE356190.1
Chlamydia psittaci WC, complete sequence	617	617	95%	2e-176	98.57%	NC_018624.1
Chlamydia psittaci 01DC12, complete sequence	617	617	95%	2e-176	98.57%	NC_019391.1
Chlamydia psittaci strain Ful127 chromosome, complete genome	617	617	95%	2e-176	98.57%	NZ_CP033059.1
Chlamydia psittaci 09DC79 CP_09DC79.contig.154, whole genome shotgun sequence	612	612	95%	1e-174	98.28%	NZ_KE359669.1
Chlamydia psittaci 09DC80 CP_09DC80.contig.3, whole genome shotgun sequence	612	612	95%	1e-174	98.28%	NZ_KE356159.1
Chlamydia psittaci 09DC78 CP_09DC78.contig.2, whole genome shotgun sequence	612	612	95%	1e-174	98.28%	NZ_KE356104.1
Chlamydia psittaci 09DC77 CP_09DC77.contig.3, whole genome shotgun sequence	612	612	95%	1e-174	98.28%	NZ_KE356032.1
Chlamydia psittaci str. Frances Contig02, whole genome shotgun sequence	612	612	95%	1e-174	98.28%	NZ_AFVM01000002.1
Chlamydia psittaci MN, complete sequence	612	612	95%	1e-174	98.28%	NC_018627.1
Chlamydia psittaci strain L99 k141_15, whole genome shotgun sequence	612	612	95%	1e-174	98.28%	NZ_JACAAQ010000021.1
Chlamydia psittaci CP3 Contig04, whole genome shotgun sequence	612	612	95%	1e-174	98.28%	NZ_AFVN01000004.1
Chlamydia psittaci NJ1, complete sequence	606	606	95%	5e-173	97.99%	NC_018626.1
Chlamydia psittaci NJ1 Contig02, whole genome shotgun sequence	606	606	95%	5e-173	97.99%	NZ_AFVK01000002.1

Alignments

Chlamydia psittaci 6BC, complete sequence
Sequence ID: **NC_017287.1** Length: 1171667 Number of Matches: 1

Range 1: 645038 to 645386

Score	Expect	Identities	Gaps	Strand	Frame
645 bits(349)	0.0()	349/349(100%)	0/349(0%)	Plus/Plus	

Features:

```
Query  15      CAGCGCTTTAGACGAATACGAGTCCTCCCTAAATCAGAATGACACACCACAAAGACCTGG  74
               ||||||||||||||||||||||||||||||||||||||||||||||||||||||||||||
Sbjct  645038  CAGCGCTTTAGACGAATACGAGTCCTCCCTAAATCAGAATGACACACCACAAAGACCTGG  645097
Query  75      CGAGTACACGGTTTCATCATCAAATGACGCAGTGTTTTTGCTCAGAGATATAGAAAAGTT  134
               ||||||||||||||||||||||||||||||||||||||||||||||||||||||||||||
Sbjct  645098  CGAGTACACGGTTTCATCATCAAATGACGCAGTGTTTTTGCTCAGAGATATAGAAAAGTT  645157
Query  135     AACAGAAGTTATTCATACACTTCACTTCACACTTCCTAGCTGGCTGATCAATGATATCAA  194
               ||||||||||||||||||||||||||||||||||||||||||||||||||||||||||||
Sbjct  645158  AACAGAAGTTATTCATACACTTCACTTCACACTTCCTAGCTGGCTGATCAATGATATCAA  645217
Query  195     AGATATCGAAAATGGCGCAAAACGTGTGCTAGAAGTTTCACAAATGCATGGTAACGATAA  254
               ||||||||||||||||||||||||||||||||||||||||||||||||||||||||||||
Sbjct  645218  AGATATCGAAAATGGCGCAAAACGTGTGCTAGAAGTTTCACAAATGCATGGTAACGATAA  645277
Query  255     TAGAACCGGATTAGGAGTCTTACAAGGGTTATTCGACAACTACTATCAAGTGCGTGATTA  314
               ||||||||||||||||||||||||||||||||||||||||||||||||||||||||||||
Sbjct  645278  TAGAACCGGATTAGGAGTCTTACAAGGGTTATTCGACAACTACTATCAAGTGCGTGATTA  645337
Query  315     CCTGAATAGTGTTTGCTATATTGGTGATGGAACAAACTGCGGAACAAGT  363
               |||||||||||||||||||||||||||||||||||||||||||||||||
Sbjct  645338  CCTGAATAGTGTTTGCTATATTGGTGATGGAACAAACTGCGGAACAAGT  645386
```

Chlamydia psittaci 6BC, complete sequence
Sequence ID: **NC_015470.1** Length: 1171660 Number of Matches: 1
Range 1: 645127 to 645475

Score	Expect	Identities	Gaps	Strand	Frame
645 bits(349)	0.0()	349/349(100%)	0/349(0%)	Plus/Plus	

Features:

```
Query  15      CAGCGCTTTAGACGAATACGAGTCCTCCCTAAATCAGAATGACACACCACAAAGACCTGG  74
               ||||||||||||||||||||||||||||||||||||||||||||||||||||||||||||
Sbjct  645127  CAGCGCTTTAGACGAATACGAGTCCTCCCTAAATCAGAATGACACACCACAAAGACCTGG  645186
Query  75      CGAGTACACGGTTTCATCATCAAATGACGCAGTGTTTTTGCTCAGAGATATAGAAAAGTT  134
               ||||||||||||||||||||||||||||||||||||||||||||||||||||||||||||
Sbjct  645187  CGAGTACACGGTTTCATCATCAAATGACGCAGTGTTTTTGCTCAGAGATATAGAAAAGTT  645246
Query  135     AACAGAAGTTATTCATACACTTCACTTCACACTTCCTAGCTGGCTGATCAATGATATCAA  194
               ||||||||||||||||||||||||||||||||||||||||||||||||||||||||||||
Sbjct  645247  AACAGAAGTTATTCATACACTTCACTTCACACTTCCTAGCTGGCTGATCAATGATATCAA  645306
Query  195     AGATATCGAAAATGGCGCAAAACGTGTGCTAGAAGTTTCACAAATGCATGGTAACGATAA  254
               ||||||||||||||||||||||||||||||||||||||||||||||||||||||||||||
Sbjct  645307  AGATATCGAAAATGGCGCAAAACGTGTGCTAGAAGTTTCACAAATGCATGGTAACGATAA  645366
Query  255     TAGAACCGGATTAGGAGTCTTACAAGGGTTATTCGACAACTACTATCAAGTGCGTGATTA  314
               ||||||||||||||||||||||||||||||||||||||||||||||||||||||||||||
Sbjct  645367  TAGAACCGGATTAGGAGTCTTACAAGGGTTATTCGACAACTACTATCAAGTGCGTGATTA  645426
Query  315     CCTGAATAGTGTTTGCTATATTGGTGATGGAACAAACTGCGGAACAAGT  363
               |||||||||||||||||||||||||||||||||||||||||||||||||
Sbjct  645427  CCTGAATAGTGTTTGCTATATTGGTGATGGAACAAACTGCGGAACAAGT  645475
```

Chlamydia psittaci 08DC60 CP_08DC60.contig.15, whole genome shotgun sequence
Sequence ID: **NZ_KE355942.1** Length: 67038 Number of Matches: 1
Range 1: 17421 to 17769

Score	Expect	Identities	Gaps	Strand	Frame
645 bits(349)	0.0()	349/349(100%)	0/349(0%)	Plus/Plus	

Features:

```
Query  15     CAGCGCTTTAGACGAATACGAGTCCTCCCTAAATCAGAATGACACACCACAAAGACCTGG  74
              ||||||||||||||||||||||||||||||||||||||||||||||||||||||||||||
Sbjct  17421  CAGCGCTTTAGACGAATACGAGTCCTCCCTAAATCAGAATGACACACCACAAAGACCTGG  17480
Query  75     CGAGTACACGGTTTCATCATCAAATGACGCAGTGTTTTTGCTCAGAGATATAGAAAAGTT  134
              ||||||||||||||||||||||||||||||||||||||||||||||||||||||||||||
Sbjct  17481  CGAGTACACGGTTTCATCATCAAATGACGCAGTGTTTTTGCTCAGAGATATAGAAAAGTT  17540
Query  135    AACAGAAGTTATTCATACACTTCACTTCACACTTCCTAGCTGGCTGATCAATGATATCAA  194
              ||||||||||||||||||||||||||||||||||||||||||||||||||||||||||||
Sbjct  17541  AACAGAAGTTATTCATACACTTCACTTCACACTTCCTAGCTGGCTGATCAATGATATCAA  17600
Query  195    AGATATCGAAAATGGCGCAAAACGTGTGCTAGAAGTTTCACAAATGCATGGTAACGATAA  254
              ||||||||||||||||||||||||||||||||||||||||||||||||||||||||||||
Sbjct  17601  AGATATCGAAAATGGCGCAAAACGTGTGCTAGAAGTTTCACAAATGCATGGTAACGATAA  17660
Query  255    TAGAACCGGATTAGGAGTCTTACAAGGGTTATTCGACAACTACTATCAAGTGCGTGATTA  314
              ||||||||||||||||||||||||||||||||||||||||||||||||||||||||||||
Sbjct  17661  TAGAACCGGATTAGGAGTCTTACAAGGGTTATTCGACAACTACTATCAAGTGCGTGATTA  17720
Query  315    CCTGAATAGTGTTTGCTATATTGGTGATGGAACAAACTGCGGAACAAGT  363
              |||||||||||||||||||||||||||||||||||||||||||||||||
```

```
Sbjct  17721  CCTGAATAGTGTTTGCTATATTGGTGATGGAACAAACTGCGGAACAAGT  17769
```

Chlamydia psittaci strain Fa An contig0013, whole genome shotgun sequence
Sequence ID: **NZ_LZRZ01000013.1** Length: 64121 Number of Matches: 1
Range 1: 490 to 838

Score	Expect	Identities	Gaps	Strand	Frame
645 bits(349)	0.0()	349/349(100%)	0/349(0%)	Plus/Plus	

Features:

```
Query  15   CAGCGCTTTAGACGAATACGAGTCCTCCCTAAATCAGAATGACACACCACAAAGACCTGG  74
            ||||||||||||||||||||||||||||||||||||||||||||||||||||||||||||
Sbjct  490  CAGCGCTTTAGACGAATACGAGTCCTCCCTAAATCAGAATGACACACCACAAAGACCTGG  549

Query  75   CGAGTACACGGTTTCATCATCAAATGACGCAGTGTTTTTGCTCAGAGATATAGAAAAGTT  134
            ||||||||||||||||||||||||||||||||||||||||||||||||||||||||||||
Sbjct  550  CGAGTACACGGTTTCATCATCAAATGACGCAGTGTTTTTGCTCAGAGATATAGAAAAGTT  609

Query  135  AACAGAAGTTATTCATACACTTCACTTCACACTTCCTAGCTGGCTGATCAATGATATCAA  194
            ||||||||||||||||||||||||||||||||||||||||||||||||||||||||||||
Sbjct  610  AACAGAAGTTATTCATACACTTCACTTCACACTTCCTAGCTGGCTGATCAATGATATCAA  669

Query  195  AGATATCGAAAATGGCGCAAAACGTGTGCTAGAAGTTTCACAAATGCATGGTAACGATAA  254
            ||||||||||||||||||||||||||||||||||||||||||||||||||||||||||||
Sbjct  670  AGATATCGAAAATGGCGCAAAACGTGTGCTAGAAGTTTCACAAATGCATGGTAACGATAA  729

Query  255  TAGAACCGGATTAGGAGTCTTACAAGGGTTATTCGACAACTACTATCAAGTGCGTGATTA  314
            ||||||||||||||||||||||||||||||||||||||||||||||||||||||||||||
Sbjct  730  TAGAACCGGATTAGGAGTCTTACAAGGGTTATTCGACAACTACTATCAAGTGCGTGATTA  789

Query  315  CCTGAATAGTGTTTGCTATATTGGTGATGGAACAAACTGCGGAACAAGT  363
            |||||||||||||||||||||||||||||||||||||||||||||||||
Sbjct  790  CCTGAATAGTGTTTGCTATATTGGTGATGGAACAAACTGCGGAACAAGT  838
```

Chlamydia psittaci strain Zo Pa contig0003, whole genome shotgun sequence
Sequence ID: **NZ_LZRY01000003.1** Length: 70118 Number of Matches: 1
Range 1: 771 to 1119

Score	Expect	Identities	Gaps	Strand	Frame
645 bits(349)	0.0()	349/349(100%)	0/349(0%)	Plus/Plus	

Features:

```
Query  15    CAGCGCTTTAGACGAATACGAGTCCTCCCTAAATCAGAATGACACACCACAAAGACCTGG  74
             ||||||||||||||||||||||||||||||||||||||||||||||||||||||||||||
Sbjct  771   CAGCGCTTTAGACGAATACGAGTCCTCCCTAAATCAGAATGACACACCACAAAGACCTGG  830

Query  75    CGAGTACACGGTTTCATCATCAAATGACGCAGTGTTTTTGCTCAGAGATATAGAAAAGTT  134
             ||||||||||||||||||||||||||||||||||||||||||||||||||||||||||||
Sbjct  831   CGAGTACACGGTTTCATCATCAAATGACGCAGTGTTTTTGCTCAGAGATATAGAAAAGTT  890

Query  135   AACAGAAGTTATTCATACACTTCACTTCACACTTCCTAGCTGGCTGATCAATGATATCAA  194
             ||||||||||||||||||||||||||||||||||||||||||||||||||||||||||||
Sbjct  891   AACAGAAGTTATTCATACACTTCACTTCACACTTCCTAGCTGGCTGATCAATGATATCAA  950

Query  195   AGATATCGAAAATGGCGCAAAACGTGTGCTAGAAGTTTCACAAATGCATGGTAACGATAA  254
             ||||||||||||||||||||||||||||||||||||||||||||||||||||||||||||
Sbjct  951   AGATATCGAAAATGGCGCAAAACGTGTGCTAGAAGTTTCACAAATGCATGGTAACGATAA  1010

Query  255   TAGAACCGGATTAGGAGTCTTACAAGGGTTATTCGACAACTACTATCAAGTGCGTGATTA  314
             ||||||||||||||||||||||||||||||||||||||||||||||||||||||||||||
Sbjct  1011  TAGAACCGGATTAGGAGTCTTACAAGGGTTATTCGACAACTACTATCAAGTGCGTGATTA  1070

Query  315   CCTGAATAGTGTTTGCTATATTGGTGATGGAACAAACTGCGGAACAAGT  363
             |||||||||||||||||||||||||||||||||||||||||||||||||
Sbjct  1071  CCTGAATAGTGTTTGCTATATTGGTGATGGAACAAACTGCGGAACAAGT  1119
```

Chlamydia psittaci strain CR009 contig0004, whole genome shotgun sequence
Sequence ID: **NZ_LZRX01000004.1** Length: 381045 Number of Matches: 1
Range 1: 317468 to 317816

Score	Expect	Identities	Gaps	Strand	Frame
645 bits(349)	0.0()	349/349(100%)	0/349(0%)	Plus/Plus	

Features:

```
Query  15      CAGCGCTTTAGACGAATACGAGTCCTCCCTAAATCAGAATGACACACCACAAAGACCTGG  74
               ||||||||||||||||||||||||||||||||||||||||||||||||||||||||||||
Sbjct  317468  CAGCGCTTTAGACGAATACGAGTCCTCCCTAAATCAGAATGACACACCACAAAGACCTGG  317527

Query  75      CGAGTACACGGTTTCATCATCAAATGACGCAGTGTTTTTGCTCAGAGATATAGAAAAGTT  134
               ||||||||||||||||||||||||||||||||||||||||||||||||||||||||||||
Sbjct  317528  CGAGTACACGGTTTCATCATCAAATGACGCAGTGTTTTTGCTCAGAGATATAGAAAAGTT  317587

Query  135     AACAGAAGTTATTCATACACTTCACTTCACACTTCCTAGCTGGCTGATCAATGATATCAA  194
               ||||||||||||||||||||||||||||||||||||||||||||||||||||||||||||
Sbjct  317588  AACAGAAGTTATTCATACACTTCACTTCACACTTCCTAGCTGGCTGATCAATGATATCAA  317647

Query  195     AGATATCGAAAATGGCGCAAAACGTGTGCTAGAAGTTTCACAAATGCATGGTAACGATAA  254
```

```
               ||||||||||||||||||||||||||||||||||||||||||||||||||||||||||||
Sbjct  317648  AGATATCGAAAATGGCGCAAAACGTGTGCTAGAAGTTTCACAAATGCATGGTAACGATAA  317707
Query  255     TAGAACCGGATTAGGAGTCTTACAAGGGTTATTCGACAACTACTATCAAGTGCGTGATTA  314
               ||||||||||||||||||||||||||||||||||||||||||||||||||||||||||||
Sbjct  317708  TAGAACCGGATTAGGAGTCTTACAAGGGTTATTCGACAACTACTATCAAGTGCGTGATTA  317767
Query  315     CCTGAATAGTGTTTGCTATATTGGTGATGGAACAAACTGCGGAACAAGT  363
               |||||||||||||||||||||||||||||||||||||||||||||||||
Sbjct  317768  CCTGAATAGTGTTTGCTATATTGGTGATGGAACAAACTGCGGAACAAGT  317816
```

Chlamydia psittaci strain Fr Da contig0005, whole genome shotgun sequence
Sequence ID: **NZ_LZSA01000005.1** Length: 64271 Number of Matches: 1
Range 1: 581 to 929

Score	Expect	Identities	Gaps	Strand	Frame
645 bits(349)	0.0()	349/349(100%)	0/349(0%)	Plus/Plus	

Features:

```
Query  15   CAGCGCTTTAGACGAATACGAGTCCTCCCTAAATCAGAATGACACACCACAAAGACCTGG  74
            ||||||||||||||||||||||||||||||||||||||||||||||||||||||||||||
Sbjct  581  CAGCGCTTTAGACGAATACGAGTCCTCCCTAAATCAGAATGACACACCACAAAGACCTGG  640
Query  75   CGAGTACACGGTTTCATCATCAAATGACGCAGTGTTTTTGCTCAGAGATATAGAAAAGTT  134
            ||||||||||||||||||||||||||||||||||||||||||||||||||||||||||||
Sbjct  641  CGAGTACACGGTTTCATCATCAAATGACGCAGTGTTTTTGCTCAGAGATATAGAAAAGTT  700
Query  135  AACAGAAGTTATTCATACACTTCACTTCACACTTCCTAGCTGGCTGATCAATGATATCAA  194
            ||||||||||||||||||||||||||||||||||||||||||||||||||||||||||||
Sbjct  701  AACAGAAGTTATTCATACACTTCACTTCACACTTCCTAGCTGGCTGATCAATGATATCAA  760
Query  195  AGATATCGAAAATGGCGCAAAACGTGTGCTAGAAGTTTCACAAATGCATGGTAACGATAA  254
            ||||||||||||||||||||||||||||||||||||||||||||||||||||||||||||
Sbjct  761  AGATATCGAAAATGGCGCAAAACGTGTGCTAGAAGTTTCACAAATGCATGGTAACGATAA  820
Query  255  TAGAACCGGATTAGGAGTCTTACAAGGGTTATTCGACAACTACTATCAAGTGCGTGATTA  314
            ||||||||||||||||||||||||||||||||||||||||||||||||||||||||||||
Sbjct  821  TAGAACCGGATTAGGAGTCTTACAAGGGTTATTCGACAACTACTATCAAGTGCGTGATTA  880
Query  315  CCTGAATAGTGTTTGCTATATTGGTGATGGAACAAACTGCGGAACAAGT  363
            |||||||||||||||||||||||||||||||||||||||||||||||||
Sbjct  881  CCTGAATAGTGTTTGCTATATTGGTGATGGAACAAACTGCGGAACAAGT  929
```

Chlamydia psittaci C6/98 CP_C6_98.contig.1, whole genome shotgun sequence
Sequence ID: **NZ_KE359922.1** Length: 1171676 Number of Matches: 1
Range 1: 977694 to 978042

Score	Expect	Identities	Gaps	Strand	Frame
645 bits(349)	0.0()	349/349(100%)	0/349(0%)	Plus/Plus	

Features:

```
Query  15      CAGCGCTTTAGACGAATACGAGTCCTCCCTAAATCAGAATGACACACCACAAAGACCTGG  74
               ||||||||||||||||||||||||||||||||||||||||||||||||||||||||||||
Sbjct  977694  CAGCGCTTTAGACGAATACGAGTCCTCCCTAAATCAGAATGACACACCACAAAGACCTGG  977753
Query  75      CGAGTACACGGTTTCATCATCAAATGACGCAGTGTTTTTGCTCAGAGATATAGAAAAGTT  134
               ||||||||||||||||||||||||||||||||||||||||||||||||||||||||||||
Sbjct  977754  CGAGTACACGGTTTCATCATCAAATGACGCAGTGTTTTTGCTCAGAGATATAGAAAAGTT  977813
Query  135     AACAGAAGTTATTCATACACTTCACTTCACACTTCCTAGCTGGCTGATCAATGATATCAA  194
               ||||||||||||||||||||||||||||||||||||||||||||||||||||||||||||
Sbjct  977814  AACAGAAGTTATTCATACACTTCACTTCACACTTCCTAGCTGGCTGATCAATGATATCAA  977873
Query  195     AGATATCGAAAATGGCGCAAAACGTGTGCTAGAAGTTTCACAAATGCATGGTAACGATAA  254
               ||||||||||||||||||||||||||||||||||||||||||||||||||||||||||||
Sbjct  977874  AGATATCGAAAATGGCGCAAAACGTGTGCTAGAAGTTTCACAAATGCATGGTAACGATAA  977933
Query  255     TAGAACCGGATTAGGAGTCTTACAAGGGTTATTCGACAACTACTATCAAGTGCGTGATTA  314
               ||||||||||||||||||||||||||||||||||||||||||||||||||||||||||||
Sbjct  977934  TAGAACCGGATTAGGAGTCTTACAAGGGTTATTCGACAACTACTATCAAGTGCGTGATTA  977993
Query  315     CCTGAATAGTGTTTGCTATATTGGTGATGGAACAAACTGCGGAACAAGT  363
               |||||||||||||||||||||||||||||||||||||||||||||||||
Sbjct  977994  CCTGAATAGTGTTTGCTATATTGGTGATGGAACAAACTGCGGAACAAGT  978042
```

Chlamydia psittaci 02DC24 CP_02DC24.contig.72, whole genome shotgun sequence
Sequence ID: **NZ_KE359643.1** Length: 1172670 Number of Matches: 1
Range 1: 319899 to 320247

Score	Expect	Identities	Gaps	Strand	Frame
645 bits(349)	0.0()	349/349(100%)	0/349(0%)	Plus/Plus	

Features:

```
Query  15      CAGCGCTTTAGACGAATACGAGTCCTCCCTAAATCAGAATGACACACCACAAAGACCTGG  74
               ||||||||||||||||||||||||||||||||||||||||||||||||||||||||||||
Sbjct  319899  CAGCGCTTTAGACGAATACGAGTCCTCCCTAAATCAGAATGACACACCACAAAGACCTGG  319958
```

```
Query  75      CGAGTACACGGTTTCATCATCAAATGACGCAGTGTTTTTGCTCAGAGATATAGAAAAGTT  134
               ||||||||||||||||||||||||||||||||||||||||||||||||||||||||||||
Sbjct  319959  CGAGTACACGGTTTCATCATCAAATGACGCAGTGTTTTTGCTCAGAGATATAGAAAAGTT  320018

Query  135     AACAGAAGTTATTCATACACTTCACTTCACACTTCCTAGCTGGCTGATCAATGATATCAA  194
               ||||||||||||||||||||||||||||||||||||||||||||||||||||||||||||
Sbjct  320019  AACAGAAGTTATTCATACACTTCACTTCACACTTCCTAGCTGGCTGATCAATGATATCAA  320078

Query  195     AGATATCGAAAATGGCGCAAAACGTGTGCTAGAAGTTTCACAAATGCATGGTAACGATAA  254
               ||||||||||||||||||||||||||||||||||||||||||||||||||||||||||||
Sbjct  320079  AGATATCGAAAATGGCGCAAAACGTGTGCTAGAAGTTTCACAAATGCATGGTAACGATAA  320138

Query  255     TAGAACCGGATTAGGAGTCTTACAAGGGTTATTCGACAACTACTATCAAGTGCGTGATTA  314
               ||||||||||||||||||||||||||||||||||||||||||||||||||||||||||||
Sbjct  320139  TAGAACCGGATTAGGAGTCTTACAAGGGTTATTCGACAACTACTATCAAGTGCGTGATTA  320198

Query  315     CCTGAATAGTGTTTGCTATATTGGTGATGGAACAAACTGCGGAACAAGT  363
               |||||||||||||||||||||||||||||||||||||||||||||||||
Sbjct  320199  CCTGAATAGTGTTTGCTATATTGGTGATGGAACAAACTGCGGAACAAGT  320247
```

Chlamydia psittaci strain 10652_placenta H5__contig_12, whole genome shotgun sequence
Sequence ID: **NZ_PJPX01000001.1** Length: 75086 Number of Matches: 1
Range 1: 10282 to 10630

Score	Expect	Identities	Gaps	Strand	Frame
645 bits(349)	0.0()	349/349(100%)	0/349(0%)	Plus/Plus	

Features:

```
Query  15     CAGCGCTTTAGACGAATACGAGTCCTCCCTAAATCAGAATGACACACCACAAAGACCTGG  74
              ||||||||||||||||||||||||||||||||||||||||||||||||||||||||||||
Sbjct  10282  CAGCGCTTTAGACGAATACGAGTCCTCCCTAAATCAGAATGACACACCACAAAGACCTGG  10341

Query  75     CGAGTACACGGTTTCATCATCAAATGACGCAGTGTTTTTGCTCAGAGATATAGAAAAGTT  134
              ||||||||||||||||||||||||||||||||||||||||||||||||||||||||||||
Sbjct  10342  CGAGTACACGGTTTCATCATCAAATGACGCAGTGTTTTTGCTCAGAGATATAGAAAAGTT  10401

Query  135    AACAGAAGTTATTCATACACTTCACTTCACACTTCCTAGCTGGCTGATCAATGATATCAA  194
              ||||||||||||||||||||||||||||||||||||||||||||||||||||||||||||
Sbjct  10402  AACAGAAGTTATTCATACACTTCACTTCACACTTCCTAGCTGGCTGATCAATGATATCAA  10461

Query  195    AGATATCGAAAATGGCGCAAAACGTGTGCTAGAAGTTTCACAAATGCATGGTAACGATAA  254
              ||||||||||||||||||||||||||||||||||||||||||||||||||||||||||||
Sbjct  10462  AGATATCGAAAATGGCGCAAAACGTGTGCTAGAAGTTTCACAAATGCATGGTAACGATAA  10521

Query  255    TAGAACCGGATTAGGAGTCTTACAAGGGTTATTCGACAACTACTATCAAGTGCGTGATTA  314
              ||||||||||||||||||||||||||||||||||||||||||||||||||||||||||||
Sbjct  10522  TAGAACCGGATTAGGAGTCTTACAAGGGTTATTCGACAACTACTATCAAGTGCGTGATTA  10581

Query  315    CCTGAATAGTGTTTGCTATATTGGTGATGGAACAAACTGCGGAACAAGT  363
              |||||||||||||||||||||||||||||||||||||||||||||||||
Sbjct  10582  CCTGAATAGTGTTTGCTATATTGGTGATGGAACAAACTGCGGAACAAGT  10630
```

Chlamydia psittaci strain 8882_foetus NODE_7, whole genome shotgun sequence
Sequence ID: **NZ_PJPZ01000009.1** Length: 64171 Number of Matches: 1
Range 1: 566 to 914

Score	Expect	Identities	Gaps	Strand	Frame
645 bits(349)	0.0()	349/349(100%)	0/349(0%)	Plus/Plus	

Features:

```
Query  15   CAGCGCTTTAGACGAATACGAGTCCTCCCTAAATCAGAATGACACACCACAAAGACCTGG  74
            ||||||||||||||||||||||||||||||||||||||||||||||||||||||||||||
Sbjct  566  CAGCGCTTTAGACGAATACGAGTCCTCCCTAAATCAGAATGACACACCACAAAGACCTGG  625

Query  75   CGAGTACACGGTTTCATCATCAAATGACGCAGTGTTTTTGCTCAGAGATATAGAAAAGTT  134
            ||||||||||||||||||||||||||||||||||||||||||||||||||||||||||||
Sbjct  626  CGAGTACACGGTTTCATCATCAAATGACGCAGTGTTTTTGCTCAGAGATATAGAAAAGTT  685

Query  135  AACAGAAGTTATTCATACACTTCACTTCACACTTCCTAGCTGGCTGATCAATGATATCAA  194
            ||||||||||||||||||||||||||||||||||||||||||||||||||||||||||||
Sbjct  686  AACAGAAGTTATTCATACACTTCACTTCACACTTCCTAGCTGGCTGATCAATGATATCAA  745

Query  195  AGATATCGAAAATGGCGCAAAACGTGTGCTAGAAGTTTCACAAATGCATGGTAACGATAA  254
            ||||||||||||||||||||||||||||||||||||||||||||||||||||||||||||
Sbjct  746  AGATATCGAAAATGGCGCAAAACGTGTGCTAGAAGTTTCACAAATGCATGGTAACGATAA  805

Query  255  TAGAACCGGATTAGGAGTCTTACAAGGGTTATTCGACAACTACTATCAAGTGCGTGATTA  314
            ||||||||||||||||||||||||||||||||||||||||||||||||||||||||||||
Sbjct  806  TAGAACCGGATTAGGAGTCTTACAAGGGTTATTCGACAACTACTATCAAGTGCGTGATTA  865

Query  315  CCTGAATAGTGTTTGCTATATTGGTGATGGAACAAACTGCGGAACAAGT  363
            |||||||||||||||||||||||||||||||||||||||||||||||||
Sbjct  866  CCTGAATAGTGTTTGCTATATTGGTGATGGAACAAACTGCGGAACAAGT  914
```

Chlamydia psittaci strain 8882_placenta H2_13, whole genome shotgun sequence
Sequence ID: **NZ_PJQA01000012.1** Length: 70480 Number of Matches: 1
Range 1: 6903 to 7251

Score	Expect	Identities	Gaps	Strand	Frame

Score	Expect	Identities	Gaps	Strand	Frame
645 bits(349)	0.0()	349/349(100%)	0/349(0%)	Plus/Plus	

Features:

```
Query  15     CAGCGCTTTAGACGAATACGAGTCCTCCCTAAATCAGAATGACACACCACAAAGACCTGG  74
              ||||||||||||||||||||||||||||||||||||||||||||||||||||||||||||
Sbjct  6903   CAGCGCTTTAGACGAATACGAGTCCTCCCTAAATCAGAATGACACACCACAAAGACCTGG  6962
Query  75     CGAGTACACGGTTTCATCATCAAATGACGCAGTGTTTTTGCTCAGAGATATAGAAAAGTT  134
              ||||||||||||||||||||||||||||||||||||||||||||||||||||||||||||
Sbjct  6963   CGAGTACACGGTTTCATCATCAAATGACGCAGTGTTTTTGCTCAGAGATATAGAAAAGTT  7022
Query  135    AACAGAAGTTATTCATACACTTCACTTCACACTTCCTAGCTGGCTGATCAATGATATCAA  194
              ||||||||||||||||||||||||||||||||||||||||||||||||||||||||||||
Sbjct  7023   AACAGAAGTTATTCATACACTTCACTTCACACTTCCTAGCTGGCTGATCAATGATATCAA  7082
Query  195    AGATATCGAAAATGGCGCAAAACGTGTGCTAGAAGTTTCACAAATGCATGGTAACGATAA  254
              ||||||||||||||||||||||||||||||||||||||||||||||||||||||||||||
Sbjct  7083   AGATATCGAAAATGGCGCAAAACGTGTGCTAGAAGTTTCACAAATGCATGGTAACGATAA  7142
Query  255    TAGAACCGGATTAGGAGTCTTACAAGGGTTATTCGACAACTACTATCAAGTGCGTGATTA  314
              ||||||||||||||||||||||||||||||||||||||||||||||||||||||||||||
Sbjct  7143   TAGAACCGGATTAGGAGTCTTACAAGGGTTATTCGACAACTACTATCAAGTGCGTGATTA  7202
Query  315    CCTGAATAGTGTTTGCTATATTGGTGATGGAACAAACTGCGGAACAAGT  363
              |||||||||||||||||||||||||||||||||||||||||||||||||
Sbjct  7203   CCTGAATAGTGTTTGCTATATTGGTGATGGAACAAACTGCGGAACAAGT  7251
```

Chlamydia psittaci strain 9945_foetus H3_12, whole genome shotgun sequence
Sequence ID: **NZ_PJPY01000006.1** Length: 76489 Number of Matches: 1
Range 1: 10347 to 10695

Score	Expect	Identities	Gaps	Strand	Frame
645 bits(349)	0.0()	349/349(100%)	0/349(0%)	Plus/Plus	

Features:

```
Query  15     CAGCGCTTTAGACGAATACGAGTCCTCCCTAAATCAGAATGACACACCACAAAGACCTGG  74
              ||||||||||||||||||||||||||||||||||||||||||||||||||||||||||||
Sbjct  10347  CAGCGCTTTAGACGAATACGAGTCCTCCCTAAATCAGAATGACACACCACAAAGACCTGG  10406
Query  75     CGAGTACACGGTTTCATCATCAAATGACGCAGTGTTTTTGCTCAGAGATATAGAAAAGTT  134
              ||||||||||||||||||||||||||||||||||||||||||||||||||||||||||||
Sbjct  10407  CGAGTACACGGTTTCATCATCAAATGACGCAGTGTTTTTGCTCAGAGATATAGAAAAGTT  10466
Query  135    AACAGAAGTTATTCATACACTTCACTTCACACTTCCTAGCTGGCTGATCAATGATATCAA  194
              ||||||||||||||||||||||||||||||||||||||||||||||||||||||||||||
Sbjct  10467  AACAGAAGTTATTCATACACTTCACTTCACACTTCCTAGCTGGCTGATCAATGATATCAA  10526
Query  195    AGATATCGAAAATGGCGCAAAACGTGTGCTAGAAGTTTCACAAATGCATGGTAACGATAA  254
              ||||||||||||||||||||||||||||||||||||||||||||||||||||||||||||
Sbjct  10527  AGATATCGAAAATGGCGCAAAACGTGTGCTAGAAGTTTCACAAATGCATGGTAACGATAA  10586
Query  255    TAGAACCGGATTAGGAGTCTTACAAGGGTTATTCGACAACTACTATCAAGTGCGTGATTA  314
              ||||||||||||||||||||||||||||||||||||||||||||||||||||||||||||
Sbjct  10587  TAGAACCGGATTAGGAGTCTTACAAGGGTTATTCGACAACTACTATCAAGTGCGTGATTA  10646
Query  315    CCTGAATAGTGTTTGCTATATTGGTGATGGAACAAACTGCGGAACAAGT  363
              |||||||||||||||||||||||||||||||||||||||||||||||||
Sbjct  10647  CCTGAATAGTGTTTGCTATATTGGTGATGGAACAAACTGCGGAACAAGT  10695
```

Chlamydia psittaci strain Ho Re lower contig0005, whole genome shotgun sequence
Sequence ID: **NZ_LZRF01000005.1** Length: 3227 Number of Matches: 1
Range 1: 180 to 528

Score	Expect	Identities	Gaps	Strand	Frame
645 bits(349)	0.0()	349/349(100%)	0/349(0%)	Plus/Plus	

Features:

```
Query  15     CAGCGCTTTAGACGAATACGAGTCCTCCCTAAATCAGAATGACACACCACAAAGACCTGG  74
              ||||||||||||||||||||||||||||||||||||||||||||||||||||||||||||
Sbjct  180    CAGCGCTTTAGACGAATACGAGTCCTCCCTAAATCAGAATGACACACCACAAAGACCTGG  239
Query  75     CGAGTACACGGTTTCATCATCAAATGACGCAGTGTTTTTGCTCAGAGATATAGAAAAGTT  134
              ||||||||||||||||||||||||||||||||||||||||||||||||||||||||||||
Sbjct  240    CGAGTACACGGTTTCATCATCAAATGACGCAGTGTTTTTGCTCAGAGATATAGAAAAGTT  299
Query  135    AACAGAAGTTATTCATACACTTCACTTCACACTTCCTAGCTGGCTGATCAATGATATCAA  194
              ||||||||||||||||||||||||||||||||||||||||||||||||||||||||||||
Sbjct  300    AACAGAAGTTATTCATACACTTCACTTCACACTTCCTAGCTGGCTGATCAATGATATCAA  359
Query  195    AGATATCGAAAATGGCGCAAAACGTGTGCTAGAAGTTTCACAAATGCATGGTAACGATAA  254
              ||||||||||||||||||||||||||||||||||||||||||||||||||||||||||||
Sbjct  360    AGATATCGAAAATGGCGCAAAACGTGTGCTAGAAGTTTCACAAATGCATGGTAACGATAA  419
Query  255    TAGAACCGGATTAGGAGTCTTACAAGGGTTATTCGACAACTACTATCAAGTGCGTGATTA  314
              ||||||||||||||||||||||||||||||||||||||||||||||||||||||||||||
Sbjct  420    TAGAACCGGATTAGGAGTCTTACAAGGGTTATTCGACAACTACTATCAAGTGCGTGATTA  479
Query  315    CCTGAATAGTGTTTGCTATATTGGTGATGGAACAAACTGCGGAACAAGT  363
              |||||||||||||||||||||||||||||||||||||||||||||||||
Sbjct  480    CCTGAATAGTGTTTGCTATATTGGTGATGGAACAAACTGCGGAACAAGT  528
```

Chlamydia psittaci strain Ho Re upper contig0003, whole genome shotgun sequence
Sequence ID: **NZ_LZRE01000003.1** Length: 381217 Number of Matches: 1
Range 1: 317561 to 317909

Score	Expect	Identities	Gaps	Strand	Frame
645 bits(349)	0.0()	349/349(100%)	0/349(0%)	Plus/Plus	

Features:

```
Query  15      CAGCGCTTTAGACGAATACGAGTCCTCCCTAATCAGAATGACACACCACAAAGACCTGG  74
               ||||||||||||||||||||||||||||||||||||||||||||||||||||||||||||
Sbjct  317561  CAGCGCTTTAGACGAATACGAGTCCTCCCTAATCAGAATGACACACCACAAAGACCTGG  317620
Query  75      CGAGTACACGGTTTCATCATCAAATGACGCAGTGTTTTTGCTCAGAGATATAGAAAAGTT  134
               ||||||||||||||||||||||||||||||||||||||||||||||||||||||||||||
Sbjct  317621  CGAGTACACGGTTTCATCATCAAATGACGCAGTGTTTTTGCTCAGAGATATAGAAAAGTT  317680
Query  135     AACAGAAGTTATTCATACACTTCACTTCACACTTCCTAGCTGGCTGATCAATGATATCAA  194
               ||||||||||||||||||||||||||||||||||||||||||||||||||||||||||||
Sbjct  317681  AACAGAAGTTATTCATACACTTCACTTCACACTTCCTAGCTGGCTGATCAATGATATCAA  317740
Query  195     AGATATCGAAAATGGCGCAAAACGTGTGCTAGAAGTTTCACAAATGCATGGTAACGATAA  254
               ||||||||||||||||||||||||||||||||||||||||||||||||||||||||||||
Sbjct  317741  AGATATCGAAAATGGCGCAAAACGTGTGCTAGAAGTTTCACAAATGCATGGTAACGATAA  317800
Query  255     TAGAACCGGATTAGGAGTCTTACAAGGGTTATTCGACAACTACTATCAAGTGCGTGATTA  314
               ||||||||||||||||||||||||||||||||||||||||||||||||||||||||||||
Sbjct  317801  TAGAACCGGATTAGGAGTCTTACAAGGGTTATTCGACAACTACTATCAAGTGCGTGATTA  317860
Query  315     CCTGAATAGTGTTTGCTATATTGGTGATGGAACAAACTGCGGAACAAGT  363
               |||||||||||||||||||||||||||||||||||||||||||||||||
Sbjct  317861  CCTGAATAGTGTTTGCTATATTGGTGATGGAACAAACTGCGGAACAAGT  317909
```

Chlamydia psittaci strain Horse_pl chromosome
Sequence ID: **NZ_CP025423.1** Length: 1169652 Number of Matches: 1
Range 1: 642914 to 643262

Score	Expect	Identities	Gaps	Strand	Frame
645 bits(349)	0.0()	349/349(100%)	0/349(0%)	Plus/Plus	

Features:

```
Query  15      CAGCGCTTTAGACGAATACGAGTCCTCCCTAATCAGAATGACACACCACAAAGACCTGG  74
               ||||||||||||||||||||||||||||||||||||||||||||||||||||||||||||
Sbjct  642914  CAGCGCTTTAGACGAATACGAGTCCTCCCTAATCAGAATGACACACCACAAAGACCTGG  642973
Query  75      CGAGTACACGGTTTCATCATCAAATGACGCAGTGTTTTTGCTCAGAGATATAGAAAAGTT  134
               ||||||||||||||||||||||||||||||||||||||||||||||||||||||||||||
Sbjct  642974  CGAGTACACGGTTTCATCATCAAATGACGCAGTGTTTTTGCTCAGAGATATAGAAAAGTT  643033
Query  135     AACAGAAGTTATTCATACACTTCACTTCACACTTCCTAGCTGGCTGATCAATGATATCAA  194
               ||||||||||||||||||||||||||||||||||||||||||||||||||||||||||||
Sbjct  643034  AACAGAAGTTATTCATACACTTCACTTCACACTTCCTAGCTGGCTGATCAATGATATCAA  643093
Query  195     AGATATCGAAAATGGCGCAAAACGTGTGCTAGAAGTTTCACAAATGCATGGTAACGATAA  254
               ||||||||||||||||||||||||||||||||||||||||||||||||||||||||||||
Sbjct  643094  AGATATCGAAAATGGCGCAAAACGTGTGCTAGAAGTTTCACAAATGCATGGTAACGATAA  643153
Query  255     TAGAACCGGATTAGGAGTCTTACAAGGGTTATTCGACAACTACTATCAAGTGCGTGATTA  314
               ||||||||||||||||||||||||||||||||||||||||||||||||||||||||||||
Sbjct  643154  TAGAACCGGATTAGGAGTCTTACAAGGGTTATTCGACAACTACTATCAAGTGCGTGATTA  643213
Query  315     CCTGAATAGTGTTTGCTATATTGGTGATGGAACAAACTGCGGAACAAGT  363
               |||||||||||||||||||||||||||||||||||||||||||||||||
Sbjct  643214  CCTGAATAGTGTTTGCTATATTGGTGATGGAACAAACTGCGGAACAAGT  643262
```

Chlamydia psittaci 02DC15, complete sequence
Sequence ID: **NC_017292.1** Length: 1172182 Number of Matches: 1
Range 1: 645625 to 645973

Score	Expect	Identities	Gaps	Strand	Frame
645 bits(349)	0.0()	349/349(100%)	0/349(0%)	Plus/Plus	

Features:

```
Query  15      CAGCGCTTTAGACGAATACGAGTCCTCCCTAATCAGAATGACACACCACAAAGACCTGG  74
               ||||||||||||||||||||||||||||||||||||||||||||||||||||||||||||
Sbjct  645625  CAGCGCTTTAGACGAATACGAGTCCTCCCTAATCAGAATGACACACCACAAAGACCTGG  645684
Query  75      CGAGTACACGGTTTCATCATCAAATGACGCAGTGTTTTTGCTCAGAGATATAGAAAAGTT  134
               ||||||||||||||||||||||||||||||||||||||||||||||||||||||||||||
Sbjct  645685  CGAGTACACGGTTTCATCATCAAATGACGCAGTGTTTTTGCTCAGAGATATAGAAAAGTT  645744
Query  135     AACAGAAGTTATTCATACACTTCACTTCACACTTCCTAGCTGGCTGATCAATGATATCAA  194
               ||||||||||||||||||||||||||||||||||||||||||||||||||||||||||||
Sbjct  645745  AACAGAAGTTATTCATACACTTCACTTCACACTTCCTAGCTGGCTGATCAATGATATCAA  645804
Query  195     AGATATCGAAAATGGCGCAAAACGTGTGCTAGAAGTTTCACAAATGCATGGTAACGATAA  254
               ||||||||||||||||||||||||||||||||||||||||||||||||||||||||||||
Sbjct  645805  AGATATCGAAAATGGCGCAAAACGTGTGCTAGAAGTTTCACAAATGCATGGTAACGATAA  645864
Query  255     TAGAACCGGATTAGGAGTCTTACAAGGGTTATTCGACAACTACTATCAAGTGCGTGATTA  314
```

```
              ||||||||||||||||||||||||||||||||||||||||||||||||||||||||||||
Sbjct  645865  TAGAACCGGATTAGGAGTCTTACAAGGGTTATTCGACAACTACTATCAAGTGCGTGATTA  645924
Query  315     CCTGAATAGTGTTTGCTATATTGGTGATGGAACAAACTGCGGAACAAGT  363
               |||||||||||||||||||||||||||||||||||||||||||||||||
Sbjct  645925  CCTGAATAGTGTTTGCTATATTGGTGATGGAACAAACTGCGGAACAAGT  645973
```

Chlamydia psittaci C19/98, complete sequence

Sequence ID: **NC_017291.1** Length: 1169374 Number of Matches: 1

Range 1: 642854 to 643202

Score	Expect	Identities	Gaps	Strand	Frame
645 bits(349)	0.0()	349/349(100%)	0/349(0%)	Plus/Plus	

Features:

```
Query  15      CAGCGCTTTAGACGAATACGAGTCCTCCCTAAATCAGAATGACACACCACAAAGACCTGG  74
               ||||||||||||||||||||||||||||||||||||||||||||||||||||||||||||
Sbjct  642854  CAGCGCTTTAGACGAATACGAGTCCTCCCTAAATCAGAATGACACACCACAAAGACCTGG  642913
Query  75      CGAGTACACGGTTTCATCATCAAATGACGCAGTGTTTTTGCTCAGAGATATAGAAAAGTT  134
               ||||||||||||||||||||||||||||||||||||||||||||||||||||||||||||
Sbjct  642914  CGAGTACACGGTTTCATCATCAAATGACGCAGTGTTTTTGCTCAGAGATATAGAAAAGTT  642973
Query  135     AACAGAAGTTATTCATACACTTCACTTCACACTTCCTAGCTGGCTGATCAATGATATCAA  194
               ||||||||||||||||||||||||||||||||||||||||||||||||||||||||||||
Sbjct  642974  AACAGAAGTTATTCATACACTTCACTTCACACTTCCTAGCTGGCTGATCAATGATATCAA  643033
Query  195     AGATATCGAAAATGGCGCAAAACGTGTGCTAGAAGTTTCACAAATGCATGGTAACGATAA  254
               ||||||||||||||||||||||||||||||||||||||||||||||||||||||||||||
Sbjct  643034  AGATATCGAAAATGGCGCAAAACGTGTGCTAGAAGTTTCACAAATGCATGGTAACGATAA  643093
Query  255     TAGAACCGGATTAGGAGTCTTACAAGGGTTATTCGACAACTACTATCAAGTGCGTGATTA  314
               ||||||||||||||||||||||||||||||||||||||||||||||||||||||||||||
Sbjct  643094  TAGAACCGGATTAGGAGTCTTACAAGGGTTATTCGACAACTACTATCAAGTGCGTGATTA  643153
Query  315     CCTGAATAGTGTTTGCTATATTGGTGATGGAACAAACTGCGGAACAAGT  363
               |||||||||||||||||||||||||||||||||||||||||||||||||
Sbjct  643154  CCTGAATAGTGTTTGCTATATTGGTGATGGAACAAACTGCGGAACAAGT  643202
```

Chlamydia psittaci 08DC60, complete sequence

Sequence ID: **NC_017290.1** Length: 1172032 Number of Matches: 1

Range 1: 645654 to 646002

Score	Expect	Identities	Gaps	Strand	Frame
645 bits(349)	0.0()	349/349(100%)	0/349(0%)	Plus/Plus	

Features:

```
Query  15      CAGCGCTTTAGACGAATACGAGTCCTCCCTAAATCAGAATGACACACCACAAAGACCTGG  74
               ||||||||||||||||||||||||||||||||||||||||||||||||||||||||||||
Sbjct  645654  CAGCGCTTTAGACGAATACGAGTCCTCCCTAAATCAGAATGACACACCACAAAGACCTGG  645713
Query  75      CGAGTACACGGTTTCATCATCAAATGACGCAGTGTTTTTGCTCAGAGATATAGAAAAGTT  134
               ||||||||||||||||||||||||||||||||||||||||||||||||||||||||||||
Sbjct  645714  CGAGTACACGGTTTCATCATCAAATGACGCAGTGTTTTTGCTCAGAGATATAGAAAAGTT  645773
Query  135     AACAGAAGTTATTCATACACTTCACTTCACACTTCCTAGCTGGCTGATCAATGATATCAA  194
               ||||||||||||||||||||||||||||||||||||||||||||||||||||||||||||
Sbjct  645774  AACAGAAGTTATTCATACACTTCACTTCACACTTCCTAGCTGGCTGATCAATGATATCAA  645833
Query  195     AGATATCGAAAATGGCGCAAAACGTGTGCTAGAAGTTTCACAAATGCATGGTAACGATAA  254
               ||||||||||||||||||||||||||||||||||||||||||||||||||||||||||||
Sbjct  645834  AGATATCGAAAATGGCGCAAAACGTGTGCTAGAAGTTTCACAAATGCATGGTAACGATAA  645893
Query  255     TAGAACCGGATTAGGAGTCTTACAAGGGTTATTCGACAACTACTATCAAGTGCGTGATTA  314
               ||||||||||||||||||||||||||||||||||||||||||||||||||||||||||||
Sbjct  645894  TAGAACCGGATTAGGAGTCTTACAAGGGTTATTCGACAACTACTATCAAGTGCGTGATTA  645953
Query  315     CCTGAATAGTGTTTGCTATATTGGTGATGGAACAAACTGCGGAACAAGT  363
               |||||||||||||||||||||||||||||||||||||||||||||||||
Sbjct  645954  CCTGAATAGTGTTTGCTATATTGGTGATGGAACAAACTGCGGAACAAGT  646002
```

Chlamydia psittaci 01DC11, complete sequence

Sequence ID: **NC_017289.1** Length: 1172197 Number of Matches: 1

Range 1: 645656 to 646004

Score	Expect	Identities	Gaps	Strand	Frame
645 bits(349)	0.0()	349/349(100%)	0/349(0%)	Plus/Plus	

Features:

```
Query  15      CAGCGCTTTAGACGAATACGAGTCCTCCCTAAATCAGAATGACACACCACAAAGACCTGG  74
               ||||||||||||||||||||||||||||||||||||||||||||||||||||||||||||
Sbjct  645656  CAGCGCTTTAGACGAATACGAGTCCTCCCTAAATCAGAATGACACACCACAAAGACCTGG  645715
Query  75      CGAGTACACGGTTTCATCATCAAATGACGCAGTGTTTTTGCTCAGAGATATAGAAAAGTT  134
               ||||||||||||||||||||||||||||||||||||||||||||||||||||||||||||
Sbjct  645716  CGAGTACACGGTTTCATCATCAAATGACGCAGTGTTTTTGCTCAGAGATATAGAAAAGTT  645775
```

```
Query  135     AACAGAAGTTATTCATACACTTCACTTCACACTTCCTAGCTGGCTGATCAATGATATCAA  194
               ||||||||||||||||||||||||||||||||||||||||||||||||||||||||||||
Sbjct  645776  AACAGAAGTTATTCATACACTTCACTTCACACTTCCTAGCTGGCTGATCAATGATATCAA  645835
Query  195     AGATATCGAAAATGGCGCAAAACGTGTGCTAGAAGTTTCACAAATGCATGGTAACGATAA  254
               ||||||||||||||||||||||||||||||||||||||||||||||||||||||||||||
Sbjct  645836  AGATATCGAAAATGGCGCAAAACGTGTGCTAGAAGTTTCACAAATGCATGGTAACGATAA  645895
Query  255     TAGAACCGGATTAGGAGTCTTACAAGGGTTATTCGACAACTACTATCAAGTGCGTGATTA  314
               ||||||||||||||||||||||||||||||||||||||||||||||||||||||||||||
Sbjct  645896  TAGAACCGGATTAGGAGTCTTACAAGGGTTATTCGACAACTACTATCAAGTGCGTGATTA  645955
Query  315     CCTGAATAGTGTTTGCTATATTGGTGATGGAACAAACTGCGGAACAAGT  363
               |||||||||||||||||||||||||||||||||||||||||||||||||
Sbjct  645956  CCTGAATAGTGTTTGCTATATTGGTGATGGAACAAACTGCGGAACAAGT  646004
```

Chlamydia psittaci strain CB3 contig4, whole genome shotgun sequence
Sequence ID: **NZ_JMEI01000004.1** Length: 62962 Number of Matches: 1
Range 1: 62045 to 62393

Score	Expect	Identities	Gaps	Strand	Frame
645 bits(349)	0.0()	349/349(100%)	0/349(0%)	Plus/Minus	

Features:

```
Query  15     CAGCGCTTTAGACGAATACGAGTCCTCCCTAAATCAGAATGACACACCACAAAGACCTGG  74
              ||||||||||||||||||||||||||||||||||||||||||||||||||||||||||||
Sbjct  62393  CAGCGCTTTAGACGAATACGAGTCCTCCCTAAATCAGAATGACACACCACAAAGACCTGG  62334
Query  75     CGAGTACACGGTTTCATCATCAAATGACGCAGTGTTTTTGCTCAGAGATATAGAAAAGTT  134
              ||||||||||||||||||||||||||||||||||||||||||||||||||||||||||||
Sbjct  62333  CGAGTACACGGTTTCATCATCAAATGACGCAGTGTTTTTGCTCAGAGATATAGAAAAGTT  62274
Query  135    AACAGAAGTTATTCATACACTTCACTTCACACTTCCTAGCTGGCTGATCAATGATATCAA  194
              ||||||||||||||||||||||||||||||||||||||||||||||||||||||||||||
Sbjct  62273  AACAGAAGTTATTCATACACTTCACTTCACACTTCCTAGCTGGCTGATCAATGATATCAA  62214
Query  195    AGATATCGAAAATGGCGCAAAACGTGTGCTAGAAGTTTCACAAATGCATGGTAACGATAA  254
              ||||||||||||||||||||||||||||||||||||||||||||||||||||||||||||
Sbjct  62213  AGATATCGAAAATGGCGCAAAACGTGTGCTAGAAGTTTCACAAATGCATGGTAACGATAA  62154
Query  255    TAGAACCGGATTAGGAGTCTTACAAGGGTTATTCGACAACTACTATCAAGTGCGTGATTA  314
              ||||||||||||||||||||||||||||||||||||||||||||||||||||||||||||
Sbjct  62153  TAGAACCGGATTAGGAGTCTTACAAGGGTTATTCGACAACTACTATCAAGTGCGTGATTA  62094
Query  315    CCTGAATAGTGTTTGCTATATTGGTGATGGAACAAACTGCGGAACAAGT  363
              |||||||||||||||||||||||||||||||||||||||||||||||||
Sbjct  62093  CCTGAATAGTGTTTGCTATATTGGTGATGGAACAAACTGCGGAACAAGT  62045
```

Chlamydia psittaci strain CB7 contig34, whole genome shotgun sequence
Sequence ID: **NZ_JMBZ01000034.1** Length: 25542 Number of Matches: 1
Range 1: 24625 to 24973

Score	Expect	Identities	Gaps	Strand	Frame
645 bits(349)	0.0()	349/349(100%)	0/349(0%)	Plus/Minus	

Features:

```
Query  15     CAGCGCTTTAGACGAATACGAGTCCTCCCTAAATCAGAATGACACACCACAAAGACCTGG  74
              ||||||||||||||||||||||||||||||||||||||||||||||||||||||||||||
Sbjct  24973  CAGCGCTTTAGACGAATACGAGTCCTCCCTAAATCAGAATGACACACCACAAAGACCTGG  24914
Query  75     CGAGTACACGGTTTCATCATCAAATGACGCAGTGTTTTTGCTCAGAGATATAGAAAAGTT  134
              ||||||||||||||||||||||||||||||||||||||||||||||||||||||||||||
Sbjct  24913  CGAGTACACGGTTTCATCATCAAATGACGCAGTGTTTTTGCTCAGAGATATAGAAAAGTT  24854
Query  135    AACAGAAGTTATTCATACACTTCACTTCACACTTCCTAGCTGGCTGATCAATGATATCAA  194
              ||||||||||||||||||||||||||||||||||||||||||||||||||||||||||||
Sbjct  24853  AACAGAAGTTATTCATACACTTCACTTCACACTTCCTAGCTGGCTGATCAATGATATCAA  24794
Query  195    AGATATCGAAAATGGCGCAAAACGTGTGCTAGAAGTTTCACAAATGCATGGTAACGATAA  254
              ||||||||||||||||||||||||||||||||||||||||||||||||||||||||||||
Sbjct  24793  AGATATCGAAAATGGCGCAAAACGTGTGCTAGAAGTTTCACAAATGCATGGTAACGATAA  24734
Query  255    TAGAACCGGATTAGGAGTCTTACAAGGGTTATTCGACAACTACTATCAAGTGCGTGATTA  314
              ||||||||||||||||||||||||||||||||||||||||||||||||||||||||||||
Sbjct  24733  TAGAACCGGATTAGGAGTCTTACAAGGGTTATTCGACAACTACTATCAAGTGCGTGATTA  24674
Query  315    CCTGAATAGTGTTTGCTATATTGGTGATGGAACAAACTGCGGAACAAGT  363
              |||||||||||||||||||||||||||||||||||||||||||||||||
Sbjct  24673  CCTGAATAGTGTTTGCTATATTGGTGATGGAACAAACTGCGGAACAAGT  24625
```

Chlamydia psittaci UGA Contig04, whole genome shotgun sequence
Sequence ID: **NZ_AWXQ01000004.1** Length: 379526 Number of Matches: 1
Range 1: 315941 to 316289

Score	Expect	Identities	Gaps	Strand	Frame
645 bits(349)	0.0()	349/349(100%)	0/349(0%)	Plus/Plus	

Features:

```
Query  15      CAGCGCTTTAGACGAATACGAGTCCTCCCTAAATCAGAATGACACACCACAAAGACCTGG  74
Sbjct  315941  CAGCGCTTTAGACGAATACGAGTCCTCCCTAAATCAGAATGACACACCACAAAGACCTGG  316000
Query  75      CGAGTACACGGTTTCATCATCAAATGACGCAGTGTTTTTGCTCAGAGATATAGAAAAGTT  134
Sbjct  316001  CGAGTACACGGTTTCATCATCAAATGACGCAGTGTTTTTGCTCAGAGATATAGAAAAGTT  316060
Query  135     AACAGAAGTTATTCATACACTTCACTTCACACTTCCTAGCTGGCTGATCAATGATATCAA  194
Sbjct  316061  AACAGAAGTTATTCATACACTTCACTTCACACTTCCTAGCTGGCTGATCAATGATATCAA  316120
Query  195     AGATATCGAAAATGGCGCAAAACGTGTGCTAGAAGTTTCACAAATGCATGGTAACGATAA  254
Sbjct  316121  AGATATCGAAAATGGCGCAAAACGTGTGCTAGAAGTTTCACAAATGCATGGTAACGATAA  316180
Query  255     TAGAACCGGATTAGGAGTCTTACAAGGGTTATTCGACAACTACTATCAAGTGCGTGATTA  314
Sbjct  316181  TAGAACCGGATTAGGAGTCTTACAAGGGTTATTCGACAACTACTATCAAGTGCGTGATTA  316240
Query  315     CCTGAATAGTGTTTGCTATATTGGTGATGGAACAAACTGCGGAACAAGT  363
Sbjct  316241  CCTGAATAGTGTTTGCTATATTGGTGATGGAACAAACTGCGGAACAAGT  316289
```

Chlamydia psittaci DD34 Contig01, whole genome shotgun sequence
Sequence ID: **NZ_AFVL01000001.1** Length: 380725 Number of Matches: 1
Range 1: 317086 to 317434

Score	Expect	Identities	Gaps	Strand	Frame
645 bits(349)	0.0()	349/349(100%)	0/349(0%)	Plus/Plus	

Features:

```
Query  15      CAGCGCTTTAGACGAATACGAGTCCTCCCTAAATCAGAATGACACACCACAAAGACCTGG  74
Sbjct  317086  CAGCGCTTTAGACGAATACGAGTCCTCCCTAAATCAGAATGACACACCACAAAGACCTGG  317145
Query  75      CGAGTACACGGTTTCATCATCAAATGACGCAGTGTTTTTGCTCAGAGATATAGAAAAGTT  134
Sbjct  317146  CGAGTACACGGTTTCATCATCAAATGACGCAGTGTTTTTGCTCAGAGATATAGAAAAGTT  317205
Query  135     AACAGAAGTTATTCATACACTTCACTTCACACTTCCTAGCTGGCTGATCAATGATATCAA  194
Sbjct  317206  AACAGAAGTTATTCATACACTTCACTTCACACTTCCTAGCTGGCTGATCAATGATATCAA  317265
Query  195     AGATATCGAAAATGGCGCAAAACGTGTGCTAGAAGTTTCACAAATGCATGGTAACGATAA  254
Sbjct  317266  AGATATCGAAAATGGCGCAAAACGTGTGCTAGAAGTTTCACAAATGCATGGTAACGATAA  317325
Query  255     TAGAACCGGATTAGGAGTCTTACAAGGGTTATTCGACAACTACTATCAAGTGCGTGATTA  314
Sbjct  317326  TAGAACCGGATTAGGAGTCTTACAAGGGTTATTCGACAACTACTATCAAGTGCGTGATTA  317385
Query  315     CCTGAATAGTGTTTGCTATATTGGTGATGGAACAAACTGCGGAACAAGT  363
Sbjct  317386  CCTGAATAGTGTTTGCTATATTGGTGATGGAACAAACTGCGGAACAAGT  317434
```

Chlamydia psittaci Cal10 cal10.assembly.2, whole genome shotgun sequence
Sequence ID: **NZ_AEZD01000002.1** Length: 382708 Number of Matches: 1
Range 1: 317191 to 317539

Score	Expect	Identities	Gaps	Strand	Frame
645 bits(349)	0.0()	349/349(100%)	0/349(0%)	Plus/Plus	

Features:

```
Query  15      CAGCGCTTTAGACGAATACGAGTCCTCCCTAAATCAGAATGACACACCACAAAGACCTGG  74
Sbjct  317191  CAGCGCTTTAGACGAATACGAGTCCTCCCTAAATCAGAATGACACACCACAAAGACCTGG  317250
Query  75      CGAGTACACGGTTTCATCATCAAATGACGCAGTGTTTTTGCTCAGAGATATAGAAAAGTT  134
Sbjct  317251  CGAGTACACGGTTTCATCATCAAATGACGCAGTGTTTTTGCTCAGAGATATAGAAAAGTT  317310
Query  135     AACAGAAGTTATTCATACACTTCACTTCACACTTCCTAGCTGGCTGATCAATGATATCAA  194
Sbjct  317311  AACAGAAGTTATTCATACACTTCACTTCACACTTCCTAGCTGGCTGATCAATGATATCAA  317370
Query  195     AGATATCGAAAATGGCGCAAAACGTGTGCTAGAAGTTTCACAAATGCATGGTAACGATAA  254
Sbjct  317371  AGATATCGAAAATGGCGCAAAACGTGTGCTAGAAGTTTCACAAATGCATGGTAACGATAA  317430
Query  255     TAGAACCGGATTAGGAGTCTTACAAGGGTTATTCGACAACTACTATCAAGTGCGTGATTA  314
Sbjct  317431  TAGAACCGGATTAGGAGTCTTACAAGGGTTATTCGACAACTACTATCAAGTGCGTGATTA  317490
Query  315     CCTGAATAGTGTTTGCTATATTGGTGATGGAACAAACTGCGGAACAAGT  363
Sbjct  317491  CCTGAATAGTGTTTGCTATATTGGTGATGGAACAAACTGCGGAACAAGT  317539
```

BLAST is a registered trademark of the National Library of Medicine

Support center Mailing list YouTube

- National Library Of Medicine
- National Institutes Of Health
- U.S. Department of Health & Human Services
- USA.gov

NCBI

National Center for Biotechnology Information, U.S. National Library of Medicine 8600 Rockville Pike, Bethesda MD, 20894 USA

Policies and Guidelines | Contact

COVID-19 Information

Public health information (CDC)

Research information (NIH)

SARS-CoV-2 data (NCBI)

Prevention and treatment information (HHS)

Español
